# Baricitinib is potentially effective in the treatment of refractory livedoid vasculopathy

**DOI:** 10.3389/fimmu.2022.1008392

**Published:** 2022-10-27

**Authors:** Yuyang Han, Ping Tu

**Affiliations:** ^1^ Department of Dermatology and Venereology, Peking University First Hospital, Beijing Key Laboratory of Molecular Diagnosis on Dermatoses, National Clinical Research Center for Skin and Immune Diseases, National Medical Products Administration (NMPA) Key Laboratory for Quality Control and Evaluation of Cosmetics, Beijing, China; ^2^ Department of Dermatology and Venereology, Shanxi Children’s Hospital and Shanxi Maternal and Child Health Hospital, Taiyuan, China

**Keywords:** livedoid vasculopathy, baricitinib, JAK inhibitor, safety, efficacy

## Abstract

**Background:**

Livedoid vasculopathy is a rare, chronic, and recurrent disease with limited effective treatments. Its etiopathogenesis remains incompletely understood. Baricitinib, a selective Janus kinase 1 and 2 inhibitor, has been used to treat rheumatoid arthritis and could reduce the disease severity in patients with livedoid vasculopathy.

**Methods:**

We retrospectively observed eight patients who received 2 mg/day of baricitinib for the treatment of refractory livedoid vasculopathy. We evaluated their clinical scores before and after treatment to determine its effectiveness and safety.

**Results:**

Improvement in livedoid vasculopathy was observed with significant regression in the clinical scores after baricitinib treatment. The mean clinical scores were 7.0 ± 1.6 and 1.4 ± 1.2 before and after baricitinib treatment, respectively (P <0.01). Furthermore, six out of the eight patients achieved a clinical score of 0 or 2 after treatment. These scores indicated remission. Clinical findings, including erythema, ulceration, and pain, improved in all the patients. The remission times ranged from 3 to 13 weeks, with a mean remission time of 7.75 ± 3.45 weeks. There were no reports of adverse events in any patient.

**Conclusions:**

Our study showed that baricitinib treatment was safe and could significantly relieve the signs and symptoms of livedoid vasculopathy. However, randomized controlled studies should be conducted to confirm these results.

## Introduction

Livedoid vasculopathy (LV) is a chronic, recurrent, occlusive cutaneous disease characterized by the presence of erythema and purpura on the legs, typically in the ankle region or on the back of the foot. Some studies have shown that many patients had peripheral neuropathy (1). This disease also causes intensely painful ulcerations and severely affects the patient’s quality of life (2). Furthermore, patients responded poorly to traditional therapy. However, because the etiopathogenesis of LV has not been established completely, effective and standard treatments remain lacking. Baricitinib is a selective Janus kinase 1 (JAK1) and 2 (JAK2) inhibitor used for the treatment of rheumatoid arthritis. In this study, we aimed to evaluate the treatment outcomes of baricitinib in refractory LV. We herein report the successful use of baricitinib in the treatment of refractory LV.

## Methods

### Patients

Patients who presented with LV to the Department of Dermatology in the Peking University First Hospital between January 2021 and January 2022 were enrolled in this study. The LV lesions in all enrolled patients were resistant to conventional treatments. The characteristics of the included patients are shown in **Table 1**. The patients were monitored monthly for side effects through complete blood cell counts, coagulation function tests, and liver and kidney function tests. All patients were followed-up from 10 to 28 weeks (average: 16.50 weeks) after treatment.

**Table 1:** Characteristics of patients treated with baricitinib for livedoid vasculopathy.

Patient No.	Age(years)/Sex	Localization	Duration of disease (months)	PreviousTherapies	Baricitinib dosage	Follow-up time (weeks)
1	36/F	Foot, ankle, and lower leg	120	c, t, tg, r, a, and en	2 mg/day	16
2	17/M	Foot, ankle, and lower leg	36	c, t, tg, r, a, and en	2 mg/day	10
3	26/F	Foot, ankle, lower leg, forearm, and hands	72	cg and t	2 mg/day	28
4	26/M	Foot, ankle, lower leg, and upper leg	9	c, cg, and cta	2 mg/day	16
5	8/M	Lower leg	7	c and cta	2 mg/day	11
6	26/F	Foot, ankle, lower leg, and forearm	24	c and t	2 mg/day	14
7	18/F	Foot, ankle, lower leg, and upper leg	12	c and cta	2 mg/day	16
8	12/F	Foot and ankle	12	c and cta	2 mg/day	16

a, aspirin; c, corticosteroid; t, thalidomide; tg, tripterygium glycosides; r, rivaroxaban; en, enoxaparin; cg, compound glycyrrhizin; cta, Chinese traditional anti-inflammatory drugs; F, female; M, male.

### Clinical assessment

The clinical score assessment comprised three domains for evaluation of the clinical severity of LV before and after treatment with baricitinib. The total score ranged from 0 to 8, and the items assessed were as follows: pain (0, none; 1, mild; 2, moderate; and 3, severe), ulceration (0, intact skin; 1, erosion; and 2, ulceration), and erythema (0, none; 1, mild; 2, moderate; and 3, severe).

We evaluated the clinical scores for each patient and also recorded the dosage and time required for achieving remission with baricitinib. Clinical scores of 2 or less indicated remission; remission was characterized by the disappearance of pain, erythema, or ulceration.

### Statistical analysis

All data analyses were performed using SPSS Statistics version 25.0 (IBM, Armonk, NY, USA). Metric data are presented as means ± standard deviation. Shapiro–Wilk tests were performed to evaluate differences in clinical scores before and after treatment. The data were in line with the normal distribution; hence, a paired *t-test* was used to test for significance. *P <*0.01 was considered significant.

## Results

**Figure 1:**
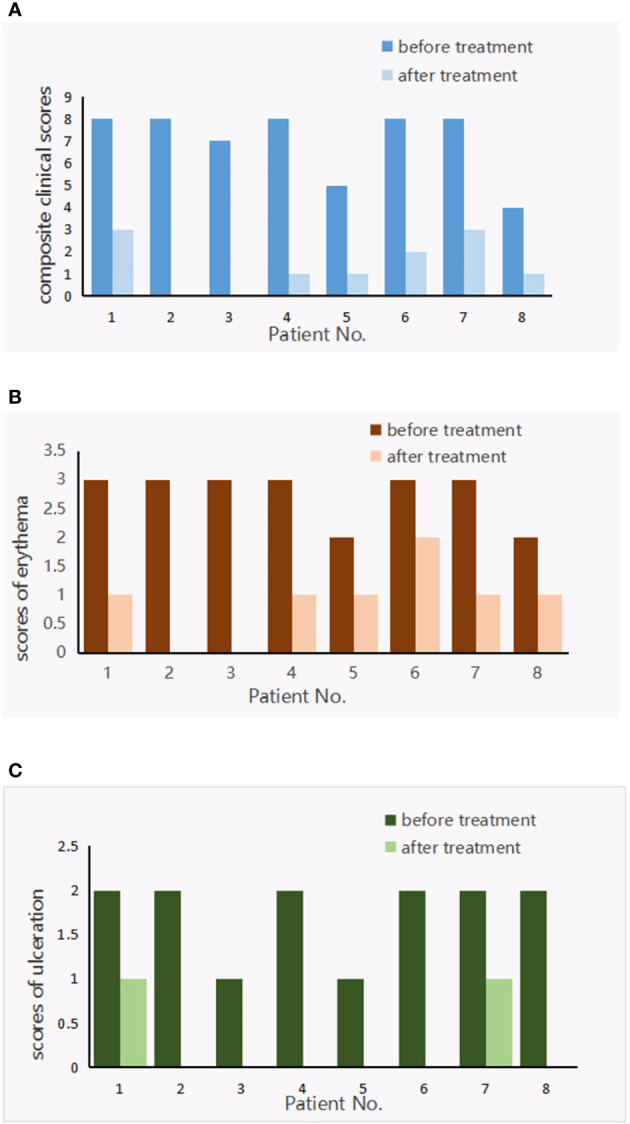
Livedoid vasculopathy composite clinical scores **(A)**, scores of erythema **(B)** and ulceration **(C)** before and after treatment with baricitinib.

**Figure 2:**
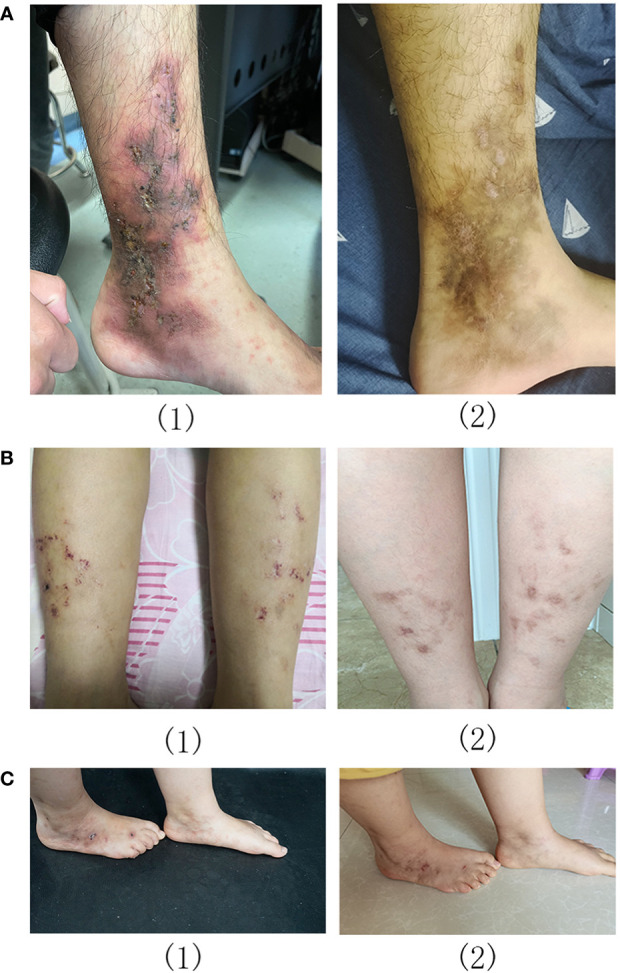
Livedoid vasculopathy before (1) and after (2) baricitinib treatment in patients 2 **(A)**, 5 **(B)**, and 8 **(C)**.

Eight patients (mean age: 21.1 ± 9.1 years) were treated with baricitinib for LV; all experienced a significant regression of LV. There was a statistically significant improvement in the clinical scores of all patients after baricitinib treatment; the mean clinical scores were 7.0 ± 1.6 and 1.4 ± 1.2 before and after baricitinib treatment, respectively (*P <*0.01). Furthermore, six out of the eight patients achieved a clinical score of 0 or 2 after treatment (**Figure 1**). All patients experienced clinical improvements, including improvements in erythema, ulceration, and pain (**Figures 1**, **2**). Patients experienced remission times ranging from 3 to 13 weeks, with a mean remission time of 7.75 ± 3.45 weeks. There were no cases of upper respiratory infections, herpes simplex virus infection, folliculitis, tuberculosis, malignant tumors, or deep vein thrombosis among our patients.

## Discussion

Baricitinib, which selectively inhibits JAK1 and JAK2, may block cytokine signaling and antagonize the effects of inflammatory cytokines (3). LV is considered an occlusive vasculopathy by most investigators. However, some findings suggest that immunological mechanisms play a potential pathogenetic role in LV development. For example, immune-mediated diseases may be associated with LV, and histological findings may include thickened blood vessel walls, homogeneous or granular immune complex deposition, fibrin and complements in the vessel wall, and minimal perivascular lymphocytic infiltrates (4, 5). In addition, the obvious effects of immunomodulatory and immunosuppressive treatments, such as colchicine, dapsone, hydroxychloroquine, anabolic steroids, and cyclosporine, suggest that the inflammatory pathway may be important for LV pathogenesis (6, 7).

The present study revealed significant remission of clinical activity in patients with refractory LV after several weeks of baricitinib treatment. Pain was greatly alleviated, and there was relief from or disappearance of the LV ulcers in these patients. The median time for patients to reach remission after baricitinib treatment was 7.75 weeks, which is slightly shorter than the 7.8 weeks reported for 50% improvement with rivaroxaban (8). However, we cannot immediately draw a definite conclusion. Further prospective research is needed to clearly compare the potential shorter time to remission of baricitinib. In our study, baricitinib treatment was administered while treatment with other drugs was stopped. This suggests that baricitinib treatment may have good efficacy without requiring additional treatments. In addition, baricitinib treatment was well-tolerated with no severe side effects reported during the follow-up examinations, suggesting that the treatment itself was safe. However, previous studies have shown that the most common adverse events associated with baricitinib treatment were nasopharyngitis, upper respiratory infections, oral herpes, and folliculitis (9). The usual treatments for LV are anticoagulants, anabolic steroids, and antiplatelets. While some patients have responded well to these treatments, they have been shown to be less effective in some patients (1). Rivaroxaban is the most commonly used anticoagulant. However, a major adverse event during treatment with rivaroxaban was bleeding (5). Compared to intravenous immunoglobulin therapy, baricitinib therapy is more convenient because it does not require injections or hospitalization and is more cost-effective. Our study included two pediatric patients, and baricitinib was safe in these pediatric patients, which is consistent with the findings of a recent report (10). The present study evaluated the clinical efficacy of baricitinib in the treatment of the largest number of LV cases in China to the best of our knowledge, through the use of clinical scores; accordingly, our findings provide reliable evidence in favor of using baricitinib for the treatment of LV.

In conclusion, our study demonstrated baricitinib as an effective treatment option for LV; this is in line with the findings from three previously published cases of LV treatment with the JAK inhibitors, tofacitinib and baricitinib (11). The safety of long-term treatment with baricitinib has been well-confirmed (12). Therefore, we believe that long-term treatment with baricitinib to prevent the flares of LV should be considered because of the safety and affordability of baricitinib. However, this study has some limitations, including its short follow-up period and relatively small sample size due to the rarity of LV. Therefore, further randomized controlled studies with larger sample sizes and longer-term tolerance studies should be conducted to confirm our results.

## Data availability statement

The original contributions presented in the study are included in the article/supplementary material. Further inquiries can be directed to the corresponding author.

## Ethics statement

The studies involving human participants were reviewed and approved by the Department of Dermatology and Venereology, Peking University First Hospital. Written informed consent to participate in this study was provided by the participants’ legal guardian/next of kin. Written informed consent was obtained from the individual(s), and minor(s)’ legal guardian/next of kin, for the publication of any potentially identifiable images or data included in this article.

## Author contributions

YH wrote the manuscript and performed the analysis. PT selected the patients, provided clinical and laboratory data, and reviewed the manuscript. All authors contributed to the article and approved the submitted version.

## Acknowledgments

We thank Shanshan Xu, for helping us to collect figures and data and Rui Huang, for sorting image.

## Conflict of interest

The authors declare that the research was conducted in the absence of any commercial or financial relationships that could be construed as a potential conflict of interest.

## Publisher’s note

All claims expressed in this article are solely those of the authors and do not necessarily represent those of their affiliated organizations, or those of the publisher, the editors and the reviewers. Any product that may be evaluated in this article, or claim that may be made by its manufacturer, is not guaranteed or endorsed by the publisher.
